# Defining the transmissible dose 50% for two pandemic influenza viruses in ferrets

**DOI:** 10.1128/jvi.01635-25

**Published:** 2026-03-11

**Authors:** C. J. Field, K. M. Septer, D. R. Patel, V. C. Weaver, D. G. Sim, K. H. Restori, M. F. Boni, T. C. Sutton

**Affiliations:** 1 Department of Veterinary and Biomedical Science, The Pennsylvania State University 8082, University Park, Pennsylvania, USA; 2 The Huck Institutes of Life Sciences, The Pennsylvania State University 124474, University Park, Pennsylvania, USA; 3 Emory Center of Excellence for Influenza Research and Response (CEIRR), University Park, Pennsylvania, USA; 4 Department of Biology, The Huck Institutes of Life Sciences, The Pennsylvania State University 124474, University Park, Pennsylvania, USA; 5 Department of Biology, Institute for Genomics and Evolutionary Medicine, Temple University 171651, Philadelphia, Pennsylvania, USA; The University of Texas Southwestern Medical Center, Dallas, Texas, USA

**Keywords:** ferret model, airborne transmission, influenza virus

## Abstract

Ferrets are widely used to model airborne transmission of influenza viruses in humans. Airborne transmission is evaluated by infecting donor ferrets with a high virus dose and monitoring transmission to contact animals sharing the same airspace. Humans can be infected with a broad range of influenza virus doses. Therefore, we evaluated the relationship between inoculation dose and transmission for two pandemic influenza viruses in ferrets. Donor ferrets were inoculated with 10^0^ to 10^6^ tissue culture infectious dose 50 (TCID_50_) of the 2009 pandemic H1N1 or 1968 pandemic H3N2 virus and were then paired with respiratory contacts. Using the proportion of donors that became infected across virus doses, we calculated the infectious dose 50 (ID_50_). Subsequently, by comparing the proportion of contacts that became infected, we calculated the transmissible dose 50% (TD_50_): the donor inoculation dose that resulted in transmission to 50% of contacts. For the 2009 pandemic H1N1 virus, the ID_50_ and TD_50_ were equivalent at <1 TCID_50_. However, for the 1968 pandemic H3N2 virus, the ID_50_ and TD_50_ were 10^0.5^ and 10^4.08^ TCID_50_ (95% CI: 10^2.34^–10^5.82^), respectively. The increased TD_50_ for the H3N2 virus was associated with significant reductions in peak viral titers and viral shedding in donors over decreasing virus inoculation doses. Collectively, these studies define a new measure of transmission that permits comparisons of transmissibility between viral strains and subtypes in ferrets. We show that the 1968 pandemic H3N2 virus has a higher TD_50_ and reduced transmissibility in ferrets relative to the 2009 pandemic H1N1 virus.

**IMPORTANCE:**

Ferrets are the gold standard animal model used to assess the transmissibility of influenza viruses. Airborne transmission is evaluated by infecting donor ferrets with a high virus dose and monitoring transmission to contact animals sharing the same airspace. However, the relationship between inoculation dose and transmission has not been evaluated in ferrets. Therefore, we performed studies evaluating airborne transmission of the 2009 pandemic H1N1 and 1968 pandemic H3N2 viruses over log scale reductions in donor inoculation doses. Using the results of these studies, we define a new measure of transmission, the transmissible dose 50%: the donor inoculation dose at which a virus is transmitted to 50% of contacts. Importantly, this metric permits the evaluation of transmissibility over a log scale. We demonstrate that the 1968 pandemic H3N2 virus has reduced transmissibility compared to the 2009 pandemic H1N1 virus in ferrets.

## INTRODUCTION

Influenza A viruses cause annual epidemics and sporadic pandemics. Currently, influenza A viruses of the H1N1 and H3N2 subtypes co-circulate in humans, and each year, the World Health Organization estimates these viruses infect up to 1 billion people worldwide, leading to 3–5 million cases of severe disease and 250,000 to 650,000 deaths (1–3). Influenza pandemics occur at irregular intervals when a virus that is antigenically novel to humans emerges from an animal reservoir (i.e., waterfowl or pigs) and then transmits efficiently from person to person via the airborne route (4). During influenza pandemics, the disease burden exceeds that of annual epidemics, and depending on the virus, disease severity can be substantial (5, 6). To study influenza virus pathogenesis and transmission, several animal models have been developed (7–9). Of these models, the ferret is the only animal model that recapitulates both disease and airborne transmission observed in humans. As a result, ferrets are the predominant animal model used to study influenza transmission, and the CDC and WHO use data on the transmissibility of emerging viruses in ferrets in their pandemic risk assessments (10, 11).

To study airborne transmission in ferrets, donor animals are typically inoculated with 10^6^ infectious units of a virus. Twenty-four hours post-infection, each donor (DR) animal is introduced into a transmission cage with a respiratory contact (RC) ferret. The transmission cages are designed such that the DR and RC share the same airspace but cannot have direct physical contact. Nasal wash samples are then collected from both the DR and RC over 10–14 days, and the samples are assayed for infectious virus. On day 14 or 21 post-pairing, blood samples are also collected from both the DR and RC to assess seroconversion (7, 8, 12). RCs are considered infected if they shed infectious virus and seroconvert. Transmission studies are often performed using three or four DR:RC pairs, and the transmission efficiency of a virus is expressed as a percentage of the contacts that become infected (i.e., two of four RCs infected equals 50% transmission efficiency). Based on studies with highly transmissible human seasonal and pandemic viruses, and poorly transmissible avian influenza viruses, viruses that transmit to 66% or greater of RCs are considered to transmit efficiently via the airborne route in ferrets and likely to have some degree of transmissibility in humans. While viruses that transmit to less than 66% of RCs are considered to have inefficient transmission in ferrets and are unlikely to exhibit transmissibility in humans (13).

Human challenge studies have found that humans can be infected with influenza viruses over a wide range of inoculation doses (reviewed in reference 14). While most challenge studies use high doses between 10^4^ and 10^7^ infectious units to ensure individuals become infected, studies evaluating the human infectious dose 50%, especially via aerosol inoculation, have found as little as 0.6–5.0 tissue culture infectious dose 50s (TCID_50_) can establish an infection in the nose of volunteers (15). However, despite the extensive use of ferrets to study influenza virus transmission, the impact of inoculation dose on transmission has not been evaluated extensively. Therefore, we conducted studies to define the relationship between infectious dose and airborne transmission in ferrets for two pandemic influenza viruses: the 1968 pandemic H3N2 virus (A/Hong Kong/1/1968) [1968 H3N2] and the 2009 pandemic H1N1 (A/California/07/2009 [H1N1pdm09]) [2009 H1N1] virus. To minimize the role of passage history, viruses were generated by reverse genetics and minimally passaged. DR ferrets were inoculated with 10–100-fold decreasing doses of virus, and transmission to RCs was monitored over 14 days. By determining the infection status of the DRs over log-scale inoculation doses, we calculated the ferret infectious dose 50% (ID_50_). Subsequently, using the proportion of RCs that became infected at each virus dose, we calculated the DR inoculation dose that resulted in transmission to 50% of contacts. We designated this as the transmissible dose 50% (TD_50_). In DR ferrets, the 2009 H1N1 and 1968 H3N2 viruses both had low ID_50_ values. As the donor inoculation dose was reduced, the 2009 H1N1 virus retained its transmissibility and had a TD_50_ equivalent to its ID_50_. In contrast, the 1968 H3N2 virus exhibited reduced transmission over decreasing inoculation doses, leading to an increase in the TD_50_.

## RESULTS

### The 2009 pandemic H1N1 virus transmits efficiently to respiratory contact ferrets over decreasing donor inoculation doses

**Fig 1:**
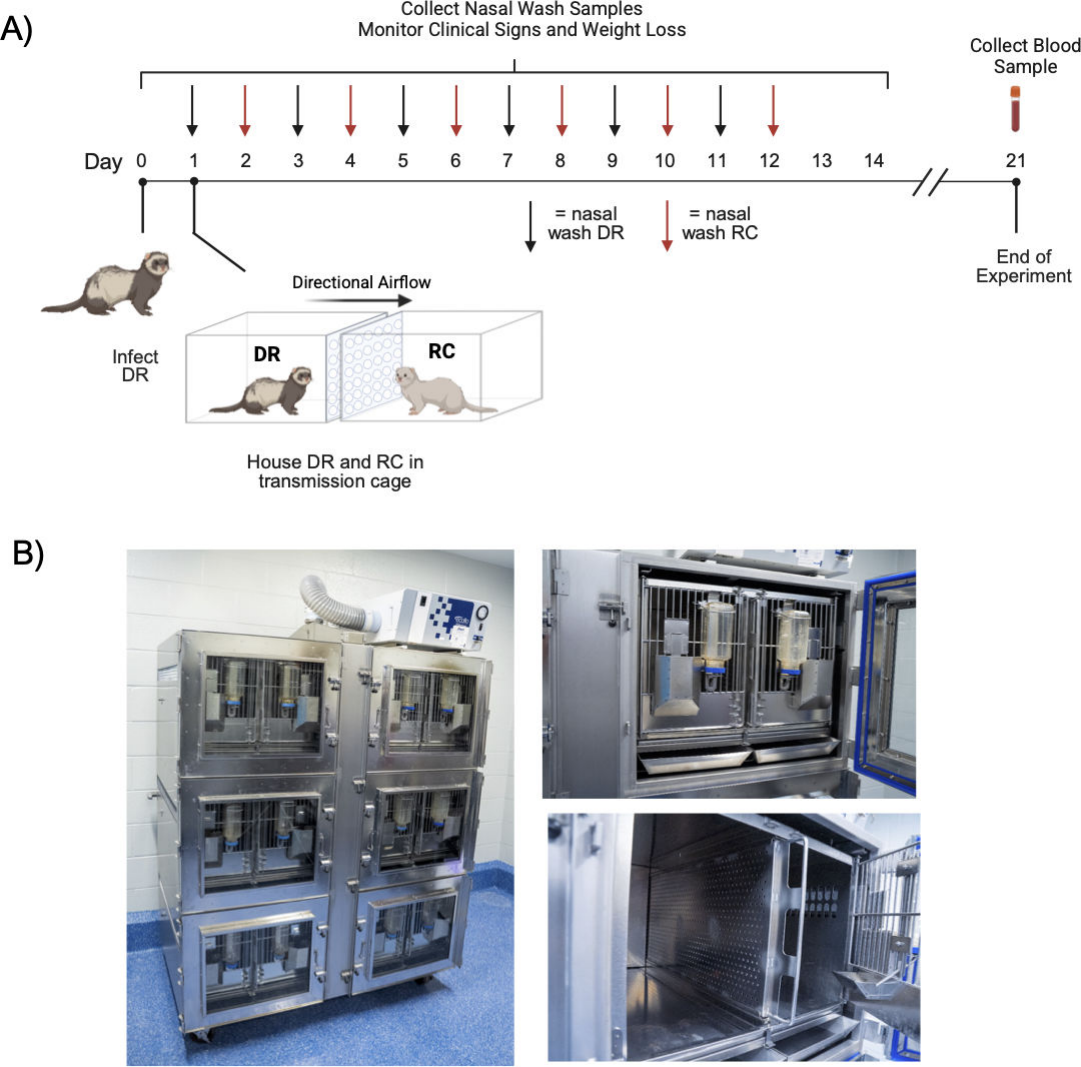
Experimental design for airborne transmission studies. (**A**) Schematic showing the timeline of ferret airborne transmission studies, including days of sampling. (**B**) Images of airborne transmission cages and custom-built ferret housing inserts. The left panel displays the airborne transmission cage with six separate compartments. Each compartment can house a DR and RC ferret. Air flows from outside of the cage toward the center of the cage, and then up a central phlegm through a HEPA filter. DR animals are housed closest to the outer wall of the cage on both the right and left sides of the rack, while RC animals are always housed next to the central phlegm. The airflow and cage are designed such that air from one compartment cannot enter another compartment. The right top panel shows the transmission compartment for a DR and RC once the outer door of the compartment is open. The right bottom panel shows the dual-layer perforated offset divider that separates the DR and RC ferrets. Each ferret has an individual feeder and water bottle. The airborne transmission cages are built by Allentown Inc., and the cages are calibrated to have 25 air changes per hour. The figure was created in BioRender. Sutton, T. (2026) https://BioRender.com/1aeunbh.

To evaluate the relationship between infectious dose and transmission, DR ferrets (*n* = 4 per inoculation dose) were inoculated with 10^6^, 10^4^, 10^2^, 10^1^, or 10^0^ TCID_50_ of recombinant 2009 H1N1. Twenty-four hours post-infection, each DR was introduced into an airborne transmission cage with a paired RC. Within the transmission cage, the animals were oriented such that airflow was directional from the DR to the RC. Nasal wash samples were then collected from the ferrets every other day for 14 days, and serum was collected from DRs and RCs on day 21 post-inoculation of the DR. A schematic of the experimental design for transmission studies and images of the transmission cages are shown in Fig. 1.

**Fig 2:**
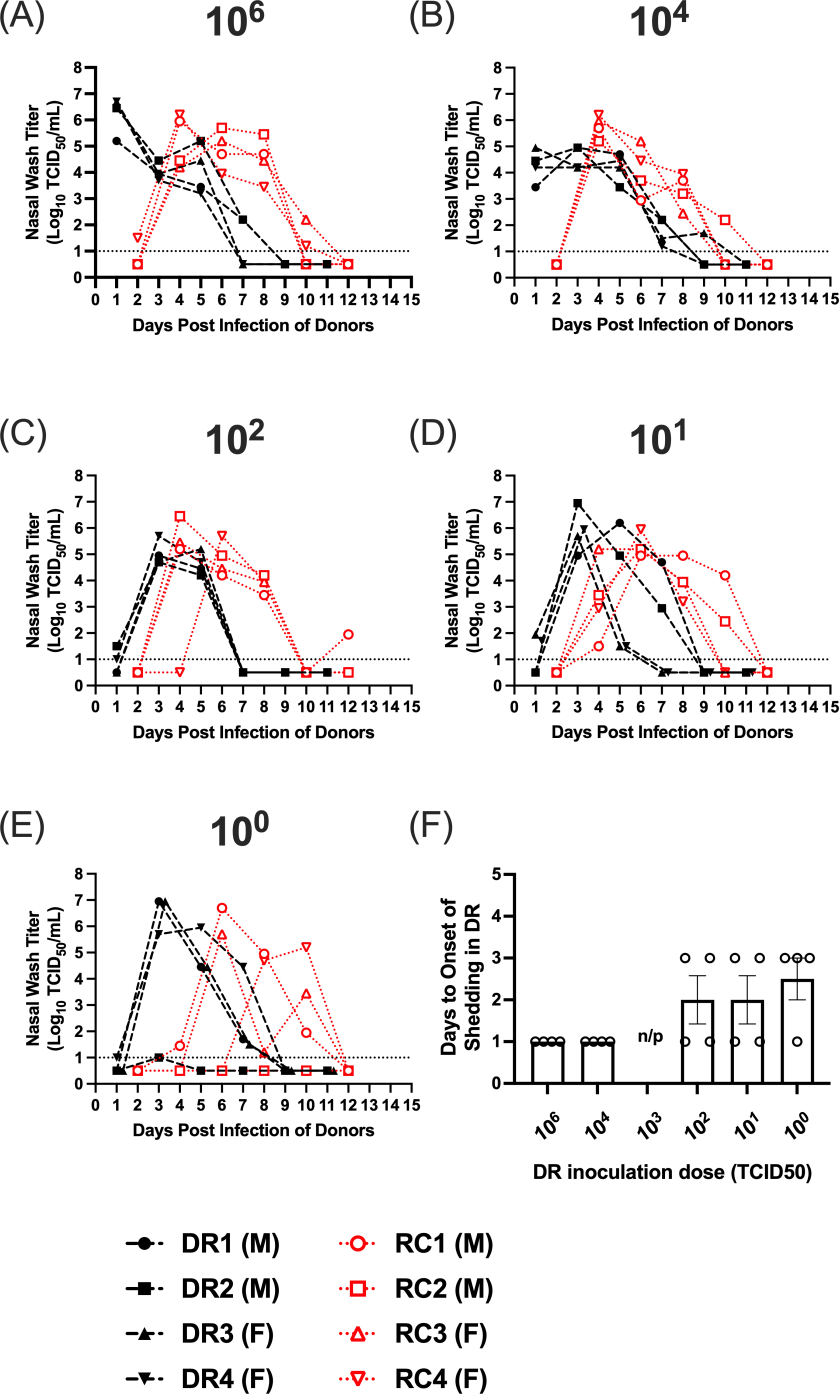
Airborne transmission of the 2009 H1N1 virus over decreasing donor inoculation doses. (**A–E**) Nasal wash titers in donors (black lines) and respiratory contacts (red lines) for decreasing donor inoculation doses. Donor inoculation dose in TCID_50_ is shown above each panel. Donor ferrets were inoculated with different doses of virus in a 1 mL volume, and 24 h post-infection, these animals were paired with respiratory contacts. Nasal wash samples were then collected from donors and contacts on alternating days. (**F**) The average number of days ± standard error to the onset of viral shedding in the DRs across virus inoculation doses. Days until the onset of shedding were compared using Kruskal-Wallis tests with a post hoc two-stage step-up procedure of Benjamini, Krieger, and Yekutieli. In panels **D** and **E**, two donors have viral shedding curves that are superimposed. These curves have been offset to facilitate visualization of the data. The dashed line denotes the limit of detection of 10^1^ TCID_50_/mL of nasal wash. M denotes male, and **F** denotes female.

During the 14-day sampling period, weight loss and clinical signs were monitored in both the DR and RC animals (Table 1; Table S1). Clinical signs were mild in all the DRs, and there were no significant differences in weight loss and changes in body temperatures across virus inoculation doses (*P* > 0.13; Kruskal-Wallis test). When RC animals became infected, clinical disease was also limited, and no significant differences were observed across DR inoculation doses (Table 1). For these studies, we used stringent criteria to define a ferret as infected, and this included virus shedding in the nasal wash on at least 1 day and seroconversion. In the DR animals, infectious virus was detected in the nasal wash by 1 day post-inoculation (dpi) for doses of 10^6^ and 10^4^ TCID_50_, and by 3 dpi for 10^2^, 10^1^, and 10^0^ TCID_50_ (Fig. 2). While not significantly different across doses, the start of viral shedding was delayed by an average of 1.5 days from the highest to lowest inoculation dose (Fig. 2F). All (100%) of DRs shed infectious virus in the nasal wash on at least 1 day for all virus doses; however, one DR, at the 10^0^ inoculation dose, had a viral titer at the limit of detection on a single day and did not seroconvert. Thus, this animal did not meet our defined criteria for infection. In all other DRs, all animals cleared the virus by 11 dpi.

**TABLE 1:** Summary of clinical signs for infected DR and RC ferrets infected with the 2009 H1N1 and 1968 H3N2 viruses

Inoculation dose	Donors		Respiratory contacts	
	Maximum change in body temperature (°C)*^a^* (mean ± SE)	Maximum weight loss (%)*^a^* (mean ± SE)	Maximum change in body temperature (°C)*^a^* (mean ± SE)	Maximum weight loss (%)*^a^* (mean ± SE)
A/California/07/2009 (H1N1pdm09)				
10^6^	0.80 ± 0.2	7.89 ± 1.5	0.93 ± 0.3	3.37 ± 1.3
10^4^	0.48 ± 0.1	6.84 ± 1.7	0.33 ± 0.1	6.10 ± 2.3
10^3^	n/p	n/p	n/p	n/p
10^2^	0.75 ± 0.5	6.30 ± 2.1	1.63 ± 0.3	8.87 ± 1.17
10^1^	0.35 ± 0.2	8.18 ± 1.6	0.93 ± 0.3	8.13 ± 1.17
10^0^	0.45 ± 0.1	7.17 ± 2.7	0.70 ± 0.3	8.63 ± 1.00
A/Hong Kong/1/1968 (H3N2)				
10^6^	0.54 ± 0.1	6.77 ± 2.2^*c*^	0.62 ± 0.4	6.05 ± 2.8
10^4^	0.48 ± 0.3	9.53 ± 4.5^*c*^	0.60 ± 0.2	7.71 ± 1.2
10^3^	0.64 ± 0.2	1.74 ± 1.0^*c*^	0.10 (only 1 RC infected)	0 (only 1 infected RC and animal gained weight)
10^2^	0.85 ± 0.3	18.6 ± 5.9*^b^*	1.00 ± 0.1 (2 RCs infected)	10.8 ± 10.8 (2 RCs infected)
10^1^	0.18 ± 0.1	1.23 ± 1.0^*c*^	No data^*d*^	1.78 (only 1 RC infected)
10^0^	0.43 ± 0.2	5.27 ± 2.6	0.60 ± 0.3	2.53 ± 0.5

^*a*^ Uninfected DR and RC animals were excluded from calculations of temperature change and weight loss. Weight loss and temperature change data for DR and RC ferrets at an inoculation dose of 10^0^ with the 1968 H3N2 virus are included for comparison of temperature changes and weight loss at other doses and between viruses.

^*b*^ Water bottles were malfunctioning, resulting in weight loss, and data were excluded from statistical analysis.

^*c*^ When maximal body weight loss was compared in DR ferrets infected with the 1968 H3N2 virus across different inoculation doses, significant differences were found via the Kruskal-Wallis test (*P *< 0.019), but due to small sample size, post hoc analyses could not resolve differences between groups.

^*d*^ No temperature data as only one RC was infected and its temperature microchip malfunctioned.

In the RCs, at donor inoculation doses of 10^6^, 10^4^, 10^2^, and 10^1^ TCID_50_, all the animals became infected, and at a DR inoculation dose of 10^0^ TCID_50_, three of four (75%) of the RCs became infected. This was confirmed by serology, and all RC ferrets that shed virus in the nasal wash seroconverted by 21 dpi of the DRs (Table 2). Infectious virus was detected in nasal washes of RCs by 4 dpi of the DRs in the 10^6^ and 10^4^ TCID_50_ groups, and by 4–8 dpi in the 10^2^, 10^1^, and 10^0^ TCID_50_ groups. All RC ferrets stopped shedding virus in the nasal wash by 12 dpi of the DRs (Fig. 2).

**TABLE 2:** Hemagglutination inhibition antibody titers in DR and RC ferrets for airborne transmission studies with the 2009 H1N1 virus^*b*^

Dose	Donor			Contact		
	Ferret number and sex (M/F)	Shed virus (Yes/No)	HI titer	Ferret number and sex (M/F)	Shed virus (Yes/No)	HI titer
10^6^	9694 (M)	Yes	1:2,560	5109 (M)	Yes	1:2,560
	5342 (M)	Yes	1:2,560	5343 (M)	Yes	1:2,560
	5428 (F)	Yes	1:2,560	5429 (F)	Yes	1:1,280
	5430 (F)	Yes	1:2,560	5439 (F)	Yes	1:1,280
10^4^	5444 (M)	Yes	1:2,560	5445 (M)	Yes	1:1,280
	5446 (M)	Yes	1:5,120	5448 (M)	Yes	1:2,560
	5432 (F)	Yes	1:2,560	5433 (F)	Yes	1:2,560
	5440 (F)	Yes	1:2,560	5441 (F)	Yes	1:2,560
10^2^	5774 (M)	Yes	1:1,280	5775 (M)	Yes	1:320
	5776 (M)	Yes	1:1,280	5777 (M)	Yes	1:1,280
	5782 (F)	Yes	1:1,280	5783 (F)	Yes	1:1,280
	5786 (F)	Yes	1:640	5787 (F)	Yes	1:1,280
10^1^	7821 (M)	Yes	1:1,280	626 (M)	Yes	1:1,920
	7822 (M)	Yes	1:640	7826 (M)	Yes	1:320
	641 (F)	Yes	1:1,280	598 (F)	Yes	1:1,280
	643 (F)	Yes	1:1,280	599 (F)	Yes	1:640
10^0^	7819 (M)	Yes	1:320	7823 (M)	Yes	1:1,280
	7820 (M)	Yes^*a*^	**<1:10**	7824 (M)	No	**<1:10**
	7814 (F)	Yes	1:240	7818 (F)	Yes	1:1,280
	7815 (F)	Yes	1:320	7684 (F)	Yes	1:1,280

^*a*^ Ferret shed virus on a single day at the limit of detection and did not seroconvert. This ferret did not meet our criteria for infection.

^*b*^ M and F denote male and female, respectively. The limit of detection was 1:10. Seroconversion was defined as a titer of 1:10 or greater. Bold font denotes animals with hemagglutination inhibition titers <1:10.

Using the number of infected DRs at each inoculation dose, we determined the ID_50_ for the 2009 H1N1 virus. As all the DRs became infected at virus inoculation doses of 10^1^ TCID_50_ and higher, and 75% of the DRs were infected at an inoculation dose of 10^0^ TCID_50_, the ID_50_ for the 2009 H1N1 virus and its confidence interval could not be estimated due to an insufficient number of uninfected ferrets. As a result, we report the ID_50_ of 2009 H1N1 as <1 TCID_50_. Similarly, using the number of RCs infected at each DR inoculation dose, we then determined the TD_50_. Likewise, as every DR that became infected with the 2009 H1N1 virus transmitted the virus to its RC, we could not obtain a reliable estimate of TD_50_ or its confidence intervals, and the TD_50_ is <1 TCID_50_. Thus, the 2009 H1N1 virus was highly transmissible even at very low inoculation doses.

### The 1968 H3N2 virus exhibited reduced and variable airborne transmission to contacts at low donor inoculation doses

**Fig 3:**
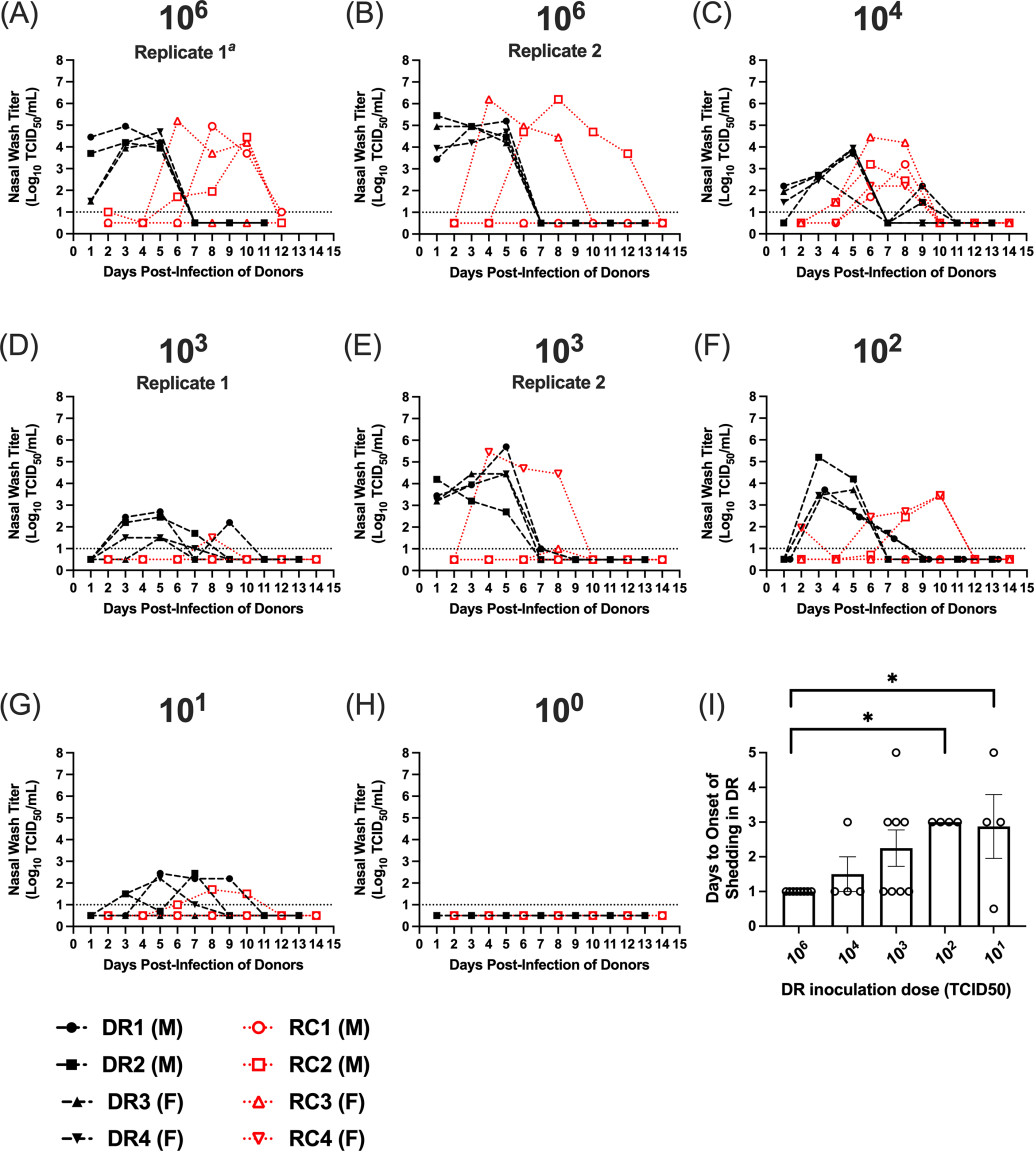
Airborne transmission of the 1968 H3N2 virus over decreasing donor inoculation doses. (**A–H**) Nasal wash titers in donors (black lines) and respiratory contacts (red lines) for transmission studies using different donor inoculation doses. Donor inoculation dose in TCID_50_ is shown above each panel, and for the 10^6^ and 10^3^ doses, two replicate studies were performed (B and E). Donor ferrets were inoculated with different doses of virus in a 1 mL volume, and 24 h post-infection, these animals were paired with respiratory contacts. Nasal wash samples were then collected from donors and contacts on alternating days. (**I**) The average number of days ± standard error to the onset of viral shedding in the DRs across virus inoculation doses. Days until the onset of shedding were compared using Kruskal-Wallis tests with a post hoc two-stage step-up procedure of Benjamini, Krieger, and Yekutieli. In panel **F**, two donors have viral shedding curves that are superimposed. These curves have been offset to facilitate visualization of the data. The dashed line denotes the limit of detection of 10^1^ TCID_50_/mL of nasal wash. M denotes male, and **F** denotes female. ^*a*^In panel **A** 10^6^ Replicate 1, the DR3 and RC3 are male; for all other panels, ferret sex is as indicated in the figure legend. *significant differences between virus doses (*P* < 0.05).

Next, we evaluated the relationship between inoculation dose and transmission for the 1968 H3N2 virus. DR ferrets were intranasally inoculated with decreasing virus doses from 10^6^ to 10^0^ TCID_50_ of the A/Hong Kong/1/1968 (H3N2) [H3N2 1968] virus. After performing transmission studies at the same doses as 2009 H1N1, transmission studies at doses of 10^3^ and 10^6^ were repeated to verify our findings. Consistent with our findings for the 2009 H1N1 virus, during the 14-day sampling period, we did not observe significant differences in the maximal changes in temperature among infected DRs at different inoculation doses or in the infected RC animals (Table 1). When we evaluated maximal weight loss, statistical testing via the Kruskal-Wallis test revealed significant differences in the DRs (*P* < 0.019), but due to small sample size, post hoc analyses could not resolve differences between groups (at an inoculation dose of 10^2^, DRs lost weight due to malfunctioning water bottles). When we evaluated weight loss in the RCs, there were no significant differences in animals that became infected across different inoculation doses (*P* > 0.52; Kruskal-Wallis test). In the DRs, infectious virus was detected in the nasal wash by 1–3 dpi at the 10^6^ and 10^4^ TCID_50_ inoculation doses, and by 3–5 dpi for 10^3^, 10^2^, and 10^1^ TCID_50_ doses (Fig. 3). On average, DRs took 2 days longer to start shedding virus in the nasal wash at an inoculation dose of 10^1^ compared to 10^6^ TCID_50_, and there was a significant delay in viral shedding for DRs in the 10^2^ and 10^1^ groups relative to the 10^6^ group (Fig. 3I). All (100%) of the DRs became infected at inoculation doses of 10^6^–10^1^ TCID_50_ and these animals also seroconverted; however, no donors inoculated with 10^0^ TCID_50_ shed virus at any time point, and these animals did not seroconvert (Fig. 3; Table 3). All infected DRs cleared the virus by 11 dpi regardless of inoculation dose.

**TABLE 3:** Hemagglutination inhibition antibody titers in DR and RC ferrets for airborne transmission studies with the 1968 H3N2 virus^*a*^

Dose	Donor			Contact		
	Ferret number and sex (M/F)	Shed virus (Yes/No)	HI titer	Ferret number and sex (M/F)	Shed virus (Yes/No)	HI titer
10^6^	1401 (M)	Yes	1:2,560	1408 (M)	Yes	1:2,560
	2120 (M)	Yes	1:640	2124 (M)	Yes	1:1,280
	2118 (M)	Yes	1:2,560	2119 (M)	No	**<1:10**
	2103 (F)	Yes	1:2,560	2104 (F)	Yes	1:2,560
	4627 (M)	Yes	1:1,280	4634 (M)	No	**<1:10**
	4629 (M)	Yes	1:1,280	4632 (M)	Yes	1:1,280
	4617 (F)	Yes	1:640	4620 (F)	No	**<1:10**
	4619 (F)	Yes	1:320	4622 (F)	Yes	1:640
10^4^	6333 (M)	Yes	1:160	6334 (M)	Yes	1:320
	6335 (M)	Yes	1:320	6336 (M)	Yes	1:640
	6321 (F)	Yes	1:160	6322 (F)	Yes	1:640
	6323 (F)	Yes	1:320	6324 (F)	Yes	1:640
10^3^	7436 (M)	Yes	1:640	7439 (M)	No	**<1:10**
	7440 (M)	Yes	n/p	7441 (M)	No	**<1:10**
	7420 (F)	Yes	1:1,280	7425 (F)	No	**<1:10**
	7428 (F)	Yes	1:160	7429 (F)	Yes	**<1:10**
	4623 (M)	Yes	1:640	4624 (M)	No	**<1:10**
	4625 (M)	Yes	1:1,280	4633 (M)	No	**<1:10**
	4613 (F)	Yes	1:1,280	4614 (F)	Yes	**<1:10**
	4615 (F)	Yes	1:1,280	4618 (F)	Yes	1:1,280
10^2^	6327 (M)	Yes	1:320	6328 (M)	No	**<1:10**
	6329 (M)	Yes	1:1,280	6330 (M)	Yes	1:640
	6313 (F)	Yes	1:320	6314 (F)	No	**<1:10**
	671 (F)	Yes	1:1,280	626 (F)	Yes	1:640
10^1^	11 (M)	Yes	1:640	7430 (M)	No	**<1:10**
	7433 (M)	Yes	1:320	7434 (M)	Yes	1:640
	7423 (F)	No	1:320	7426 (F)	No	**<1:10**
	7427 (F)	Yes	1:480	007 (F)	No	**<1:10**
10^0^	005 (M)	No	<1:10	7442 (M)	No	**<1:10**
	7437 (M)	No	<1:10	7443 (M)	No	**<1:10**
	7418 (F)	No	<1:10	7422 (F)	No	**<1:10**
	7419 (F)	No	<1:10	7424 (F)	No	**<1:10**

^*a*^ M and F denote male and female, respectively. The limit of detection was 1:10. Bold font denotes animals with hemagglutination inhibition titers <1:10. n/p denotes not performed as the serum sample was lost during processing. Seroconversion was defined as a titer of 1:10 or greater.

At an inoculation dose of 10^6^ TCID_50_, three of four (75%) and two of four (50%) RCs shed infectious virus and seroconverted in the two replicate studies, respectively (Fig. 3A and B). Thus, a total of five of eight RCs (62.5%) became infected at a DR inoculation dose of 10^6^. When DRs were inoculated with 10^4^ TCID_50_ of virus, all four RCs (100%) shed virus and seroconverted (Table 3). This was the only inoculation dose for the 1968 H3N2 virus, where the virus was transmitted to all the RCs. For the replicate studies with a DR inoculation dose of 10^3^, two of four and one of four RCs shed infectious virus in the two replicate studies; however, neither of the RCs in the first replicate seroconverted, and these animals only shed low titers of virus on a single day. In the second replicate, the single RC that shed virus did seroconvert. Thus, a total of one of eight RCs became infected (12.5%) (Fig. 3D and E; Table 3). When the DR inoculation dose was further reduced to 10^2^ and 10^1^ TCID_50_, 50% and 25% of RCs shed virus and seroconverted, respectively (Table 3). The virus was cleared from the nasal wash by 12 dpi for all RCs. At a DR inoculation dose of 10^0^ TCID_50_, none of the RCs became infected, consistent with a lack of viral shedding from their DRs.

**Fig 4:**
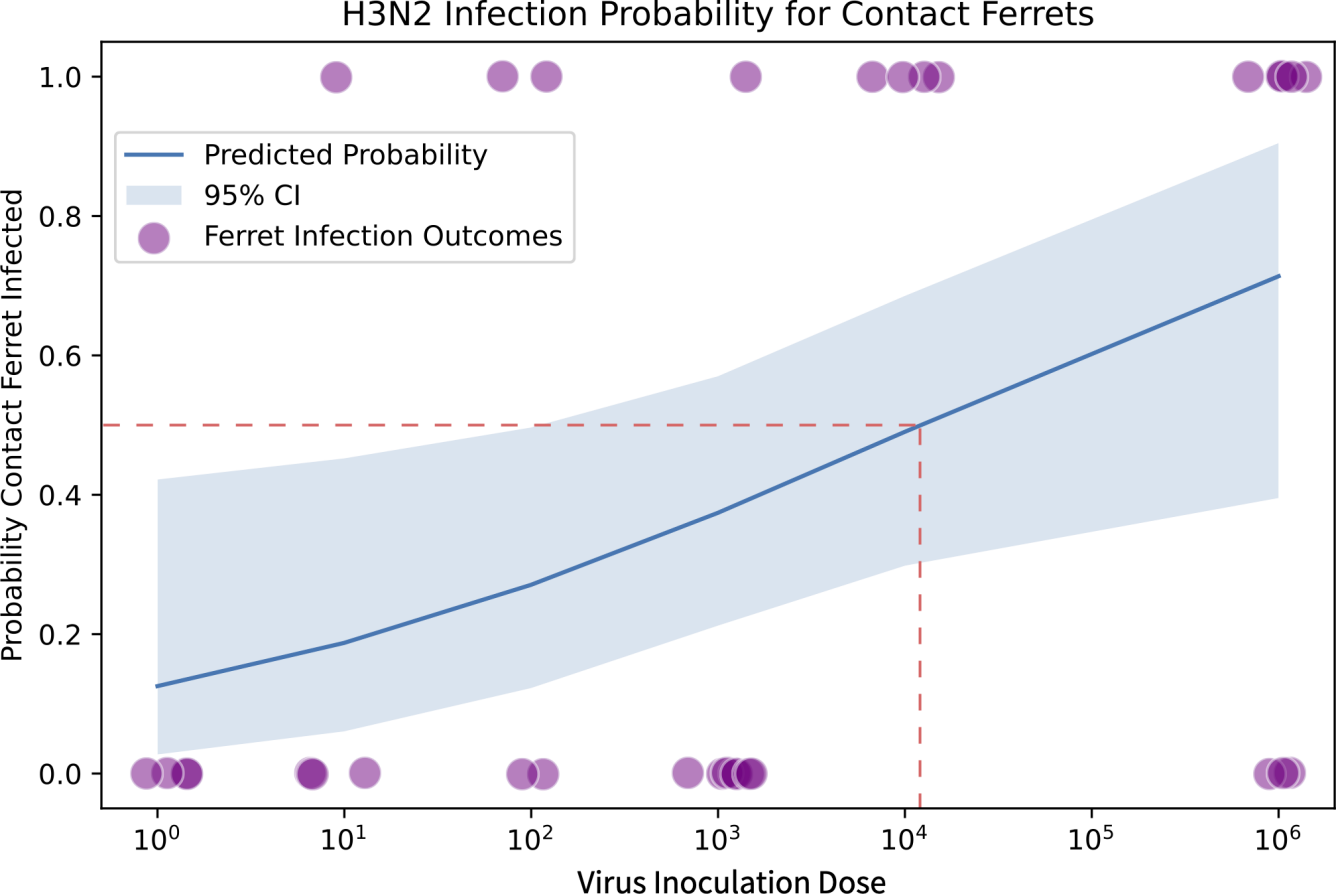
Logistic regression of 1968 H3N2 infection outcomes in respiratory contact ferrets over virus inoculation doses from 10^0^ to 10^6^ infectious units in donor ferrets. Logistical regression was performed on ferret transmission outcomes in the respiratory contacts for the 1968 H3N2 virus. The blue line shows the predicted probability of transmission to a single contact ferret based on virus inoculation dose in TCID_50_, and the blue-shaded region shows the 95% confidence interval of the predicted mean probability. Purple dots show the individual ferret infection outcomes, with zero indicating not infected and one indicating infected. The red dashed line shows the transmissible dose 50 (TD_50_).

Using the criteria of viral shedding combined with seroconversion to define an animal as infected, we calculated the ID_50_ and TD_50_ for the 1968 H3N2 virus. The ID_50_ in DRs was 10^0.5^ TCID_50_, and the TD_50_ in RCs was 10^4.08^ TCID_50_ (95% CI: 10^2.34^–10^5.82^) (Fig. 4). This ID_50_ is similar to the 2009 H1N1 virus; however, the TD_50_ is 3 orders of magnitude higher (Table 4). Therefore, while both the 2009 H1N1 and 1968 H3N2 viruses have low ID_50_ values, these viruses exhibit differing degrees of transmissibility, with the 1968 H3N2 virus having more variable and reduced transmission at low inoculation doses in ferrets (compare Fig. 2 vs Fig. 3, Table 4). Excluding the 10^3^-dose (as this was only done for 1968 H3N2), a total of 20 contact ferrets for 2009 H1N1 and 24 contact ferrets for 1968 H3N2 had binary outcomes of successful infection or not. Nonparametric statistical tests on the paired results at each dose show that H1N1 is likely to be more transmissible to contact ferrets than H3N2, via a Wilcoxon signed-rank test (*P* = 0.066) or a simple randomization test of the two sub-type labels (*P* = 0.042).

**TABLE 4:** Summary of airborne transmission studies in ferrets using the 2009 H1N1 and 1968 H3N2 viruses^*c*^^,^^*d*^

Donor inoculation dose	A/California/07/2009 (H1N1pdm09)		A/Hong Kong/1/1968 (H3N2)	
	% Donors infected^*a*^	% Contacts infected	% Donors infected^*a*^*^,b^*	% Contacts infected
10^6^	100	100	100	62.5
10^4^	100	100	100	100
10^3^	n/p	n/p	100	12.5
10^2^	100	100	100	50
10^1^	100	100	100	25
10^0^	75	75	0	0
ID_50_ or TD_50_	**ID_50_ < 1**	**TD_50_ < 1**	**ID_50_ 10^0.5^**	**TD_50_ 10^4.08^**

^*a*^ Subset of the ID_50_ data was previously reported in reference 16.

^*b*^ Due to the distribution of data, confidence intervals could not be calculated and the ID_50_ is between 10^0^ and 10^1^.

^*c*^ Ferrets were defined as infected when infectious virus was recovered in the nasal wash on at least 1 day and the animals developed HI titers above the limit of detection (i.e., 1:10 or greater).

^*d*^ Percentage of donors and respiratory contacts that became infected are described for each donor inoculation dose performed for each virus. The ID_50_ and TD_50_ are calculated at the bottom of the table and are shown in bold. n/p = not performed.

### The 2009 H1N1 virus replicated to similar peak titers across inoculation doses, while the 1968 H3N2 virus exhibited reduced replication and overall shedding at lower inoculation doses

**Fig 5:**
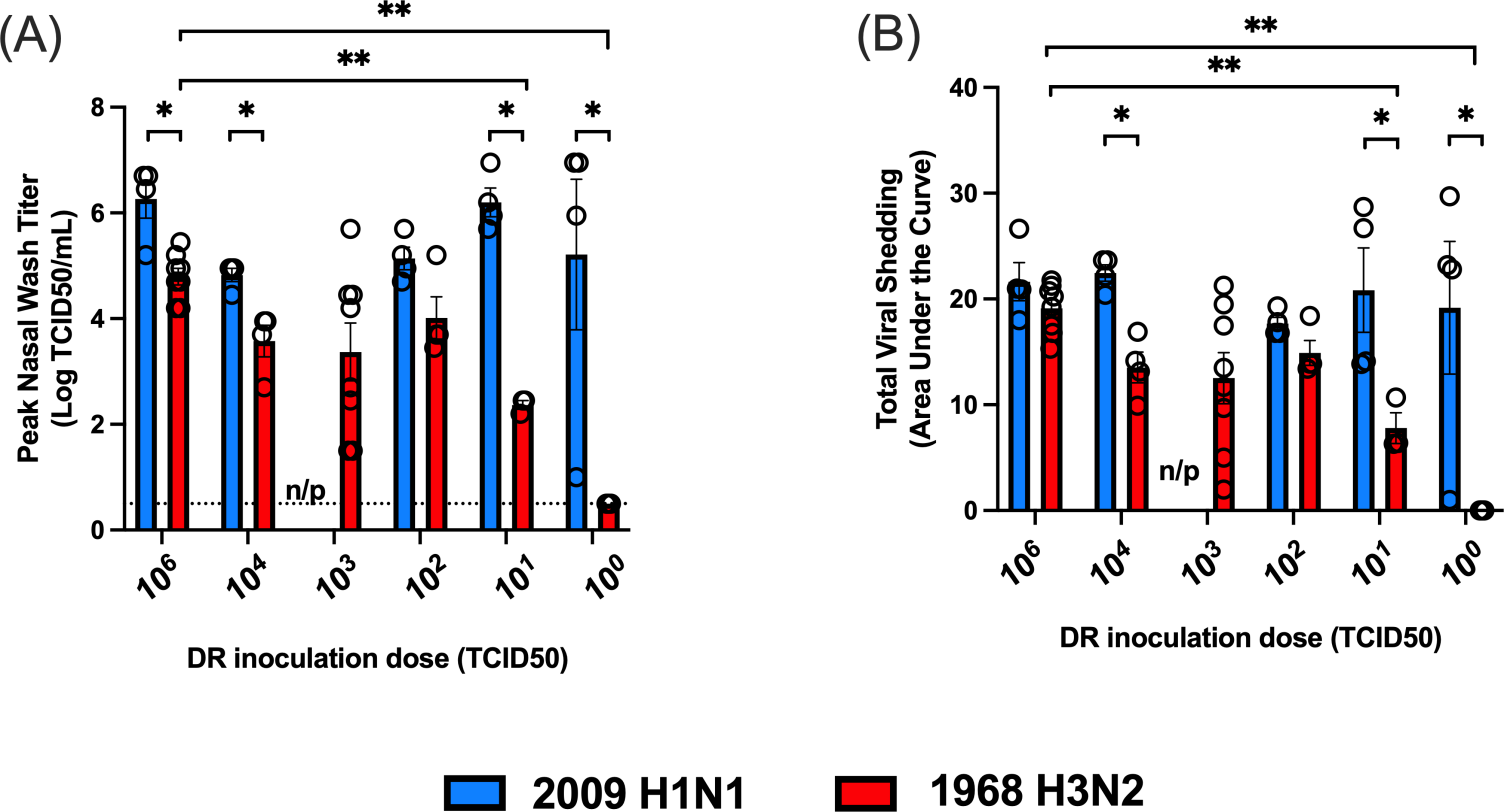
Analysis of peak viral titers and total viral shedding in donor ferrets across decreasing virus inoculation doses for the 2009 H1N1 and 1968 H3N2 viruses. (**A** and **B**) Peak viral titers and total virus shedding, respectively, for DR ferrets infected with the 2009 H1N1 or 1968 H3N2 viruses over decreasing virus inoculation doses. Blue and red bars represent the 2009 H1N1 and 1968 H3N2 viruses, respectively. Total viral shedding was determined via the area under the curve for the nasal wash titer curve. Peak titers and total viral shedding were compared across virus doses for a given virus strain, and between virus strains for a specific virus dose. *Significant differences between virus strains at the indicated dose (*P* < 0.05). **Significant differences between virus doses for virus strain (*P* < 0.05). n/p denotes not performed because this virus dose was not tested in ferrets for the 2009 H1N1 virus. For comparisons between DR inoculation doses for a specific virus strain, Kruskal-Wallis tests were performed with a post hoc two-stage step-up procedure of Benjamini, Krieger, and Yekutieli. For comparisons between virus strains at a specific dose, Mann-Whitney U tests were performed.

To determine if differences in the TD_50_ were associated with differences in viral replication kinetics, we compared peak titers and total viral shedding in the DRs across inoculation doses for the 2009 H1N1 and 1968 H3N2 viruses. We found DRs infected with the 2009 H1N1 virus had significantly higher peak titers than DRs infected with the H3N2 virus at inoculation doses of 10^6^, 10^4^, 10^1^, and 10^0^ TCID_50_ (Fig. 5A). Moreover, peak titers for the 2009 H1N1 virus were similar over decreasing virus inoculation doses. In contrast, peak titers in DRs infected with the 1968 H3N2 virus were reduced at lower inoculation doses. Peak 1968 H3N2 virus titers for the 10^1^ TCID_50_ group were significantly lower than those for animals inoculated with 10^6^ TCID_50_, and DRs inoculated with 10^0^ TCID_50_ did not become infected.

To quantify total virus shedding, we calculated the area under the viral titer curve for each infected DR. We found that DRs infected with the 2009 H1N1 virus shed more virus than those infected with the H3N2 virus (Fig. 5B). This was statistically significant for inoculation doses of 10^4^, 10^1^, and 10^0^ TCID_50_. There was also a significant reduction in the total amount of virus shed in DRs infected with 10^1^ TCID_50_ of the H3N2 virus relative to those infected with 10^6^ TCID_50_, and no ferrets shed virus at the 10^0^ TCID_50_ dose. Therefore, the higher TD_50_ for the 1968 H3N2 virus was associated with reduced peak titers and virus shedding compared to the 2009 H1N1 virus; although these metrics were not significantly different at all doses tested.

## DISCUSSION

Clinical and epidemiological studies have reported that humans are susceptible to influenza virus infection over a range of virus doses, with reports of doses as low as 0.6 and up to 10^7^ infectious units being sufficient to establish infection (14, 15). In contrast, the ferret transmission model typically utilizes a high inoculation dose (7, 12). This high-dose inoculum is often used to ensure the ferrets become infected, thereby permitting the evaluation of clinical illness (7). However, the impact of inoculation dose and the relationship between inoculation dose and transmission in ferrets has not been studied extensively. Therefore, we inoculated DR ferrets with 10–100-fold decreasing doses of the 2009 H1N1 or 1968 H3N2 virus and assessed transmission to RCs. In the DR ferrets, both the 2009 H1N1 and 1968 H3N2 viruses had a low ID_50_ of <1 and 10^0.5^ TCID_50_, respectively. We previously published a subset of this ID_50_ data as part of our risk assessment studies on an H5N1 virus isolated from mink (16). Here, we build upon these studies and assess transmission from these inoculated ferrets to respiratory contact animals. We found that despite both viruses having a low ID_50_, the viruses differed in their transmissibility. To capture differences in transmissibility, we defined a new metric, the transmissible dose 50%, which is the DR inoculation dose that results in transmission to 50% of RCs. We found the 2009 H1N1 virus had a low TD_50_ of <1 TCID_50_, which is comparable to the ID_50_ for this virus. This indicates that for the 2009 H1N1 virus, if a DR ferret becomes infected, it is likely to transmit the virus to its RC. In contrast, the TD_50_ for the 1968 H3N2 virus was several orders of magnitude higher and was 10^4.08^ TCID_50_. Therefore, the 1968 H3N2 virus required much higher inoculation doses to consistently support transmission.

We then analyzed the peak titers and total viral shedding across DR inoculation doses. We found the 2009 H1N1 virus consistently replicated to comparable peak titers and was shed at similar levels regardless of inoculation dose. These findings suggest that if a ferret becomes infected with the 2009 H1N1 virus, regardless of inoculation dose, the virus will exhibit similar replication kinetics and transmit onward to contact animals. In contrast, there were reductions in peak titers and total virus shedding for the 1968 H3N2 virus as the DR inoculation dose was reduced, and these reductions in replication were associated with reduced transmission to contact animals. Experimental studies have evaluated the extent to which different influenza A virus strains establish an infection via the delivery of viral gene segments in relatively few particles carrying up to eight required segments or multiple particles carrying relatively few segments (17–19). This property is referred to as a reliance on multiple infections. These studies have demonstrated that a seasonal H3N2 virus (A/Panama/2007/1999 [H3N2]) has high reliance on multiple infection, while the 2009 pandemic H1N1 virus has low reliance on multiple infection. Thus, we speculate that as the virus inoculation dose is reduced for the 1968 H3N2 virus, due to a high reliance on multiple infection, the virus has reduced ability to replicate, which reduces peak titers and onward transmission. In contrast, for the 2009 H1N1 virus, due to a low reliance on multiple infection, even at low inoculation doses, the virus can replicate to high titers and transmission onward. This requires further evaluation using other representative 1968 H3N2 and 2009 H1N1 pandemic strains and more contemporary H3N2 and H1N1 viruses.

Our findings that ferrets can be infected with low doses of the 2009 H1N1 and 1968 H3N2 viruses are consistent with those reported by other groups (20–23). When the ID_50_ was determined in ferrets for several swine H1N1 reassortant viruses and the 2009 pandemic H1N1 virus, the ID_50_ for these viruses varied from <10 to 18 TCID_50_ (20). Our findings are also consistent with studies evaluating replication of the A/California/04/2009 (H1N1pdm09) isolate at reduced inoculation doses. In these studies, ferrets were inoculated with doses of 10^6^, 10^4^, and 10^2^ TCID_50_ of virus (21). At each virus dose, all ferrets became infected, and peak viral replication in animals inoculated with 10^2^ TCID_50_ was delayed. Moreover, across the three inoculation doses, the A/California/04/2009 (H1N1pdm09) virus replicated to similar peak titers. In separate studies, ferrets were infected with 10^2^ or 10^5^ TCID_50_ of the A/California/07/2009 (H1N1pdm09) isolate, and replication kinetics were monitored over 9 days. Consistent with our findings at an inoculation dose of 10^4^ TCID_50_ (Fig. 2B), inoculation with 10^5^ TCID_50_ resulted in a plateau in virus titers from days 1 to 5, followed by a decline in titers on days 7 and 9. At an inoculation dose of 10^2^ TCID_50_, replication kinetics mirrored those of our studies (Fig. 2C): shortly after virus inoculation, there was minimal viral shedding, followed by replication to peak titers between 3 and 5 dpi, and then viral clearance by 9 dpi (22).

When the ID_50_ was determined in ferrets for a seasonal H3N2 virus, A/Victoria/3/1975 (H3N2), it was 10^0.66^ or five plaque-forming units (pfu) (23). This is directly comparable to the ID_50_ for the 1968 H3N2 virus used in our studies. In studies with the A/Victoria/3/1975 (H3N2) virus, the replication kinetics were analyzed in pairs of ferrets infected with 10^4^, 10^2^, and 10^1^ pfu of virus. At the lower inoculation doses of 10^2^ and 10^1^, viral titers peaked on day 2 or 3 and then declined steadily over the next 5 days. At an inoculation dose of 10^4^ pfu, viral titers peaked on day 2, declined to near the limit of detection on day 3, and were then variable until day 7, when the virus was cleared. These replication kinetics differ from those we observed for the 1968 H3N2 virus. For the 1968 H3N2 virus, at a DR inoculation dose of 10^6^, viral titers were constant between 1 and 5 dpi, and the virus was cleared between 5 and 7 dpi. At lower inoculation doses (i.e., 10^4^–10^1^) of the 1968 H3N2 virus, viral titers in individual DRs were highly variable. It is unclear why the replication kinetics differed between the 1968 H3N2 virus and the A/Victoria/3/1975 (H3N2) strain. This may be due to differences in ferret sampling procedures or may reflect further mammalian adaptation of the A/Victoria/3/1975 (H3N2) strain resulting from extensive circulation in humans.

To our knowledge, we are the first to assess airborne transmission of two seasonal influenza viruses in parallel over log-fold decreasing DR inoculation doses in ferrets. Our findings of 100% and 62.5% transmission efficiency for the 2009 H1N1 and 1968 H3N2 viruses, respectively, at a DR inoculation dose of 10^6^ TCID_50_ are within the range of transmission efficiency reported in prior studies; although seasonal H3N2 viruses are often reported to transmit to between 66% and 100% of RCs (reviewed in reference 13). In a study comparing airborne transmission after intranasal inoculation with a seasonal H3N2 virus, A/Panama/2007/1999 (H3N2), ferrets were infected with high, intermediate, and low doses consisting of approximately 2, 23, and 100–227 plaque-forming units per ferret. After virus inoculation, these animals were then used as DRs to two RCs (24). All the DR ferrets shed replicating virus in the nasal wash, and in the high dose group, two of two RCs became infected. In contrast, for both the low and intermediate doses, the virus did not transmit to RCs. Thus, in both these and our studies, the same pattern of reduced transmission for an H3N2 virus was observed as the inoculation dose was reduced; however, the virus doses used with the A/Panama/2007/1999 (H3N2) virus were much lower than in our study. It is possible that during circulation in humans, the A/Panama/2007/1999 (H3N2) virus evolved to replicate more efficiently in mammalian cells, thereby facilitating transmission at low inoculation doses.

Importantly, direct contact transmission of seasonal H3N2 viruses has also been evaluated at reduced DR inoculation doses in ferrets and guinea pigs (23, 25). In direct contact transmission studies in ferrets, DR ferrets were infected with 10^1^ pfu of the A/Victoria/3/1975 (H3N2) virus and co-housed with a contact animal 24 h post-infection. In these experiments, all the DRs were productively infected, and they transmitted the virus to 100% of contacts (23). Similarly, when guinea pigs were inoculated with 10^3^, 10^2^, and 10^1^ pfu of the A/Panama/2007/1999 (H3N2) virus and co-housed with contacts, 100% of the contacts became infected (25). In our studies, the 1968 H3N2 virus transmitted to 12.5%, 50%, and 25% of respiratory contacts at low inoculation doses of 10^3^, 10^2^, and 10^1^ TCID_50_, respectively. These discrepancies in transmission are most likely due to airborne transmission imposing a more stringent bottleneck on the number of virions transmitted to contact animals relative to direct contact transmission (26).

Collectively, our studies provide a new approach and metric to study airborne transmission in ferrets. We show that while both the 2009 H1N1 and 1968 H3N2 viruses have low ID_50_s, these viruses exhibit differing degrees of transmissibility. For the 2009 H1N1 virus, all infected DRs, regardless of inoculation dose, transmitted the virus to their RCs, and the ID_50_ and TD_50_ were both very low. Moreover, across DR inoculation doses, the 2009 H1N1 virus replicated to similar peak titers, and similar amounts of virus were shed. In contrast, for the 1968 H3N2 virus, as the DR inoculation dose decreased, peak titers and total virus shedding were reduced, resulting in reduced transmission and an increased TD_50_. Importantly, the use of TD_50_ has the advantage of permitting comparisons of transmissibility over a continuous scale. This contrasts with the conventional approach of defining transmissibility in 25%–33% increments, with transmission to greater than 66% of contacts considered efficient. For example, using the conventional high-dose inoculation approach, the transmission efficiency of the 1968 H3N2 and 2009 H1N1 viruses would have been 62.5% and 100% versus TD_50_ values of 10^4.08^ and <1, respectively. In the future, the TD_50_ metric could be applied in several different contexts. This could include comparing the TD_50_ of seasonal H3N2 viruses collected since 1968 to determine if these viruses have acquired enhanced transmissibility during their circulation in humans. Moreover, it will be valuable to determine the TD_50_ of other pandemic viruses, such as representative strains of the 1957 H2N2 and 1918 H1N1 viruses. The TD_50_ of pandemic viruses could then be compared to other zoonotic or emerging viruses that exhibit airborne transmission in ferrets. This may reveal distinct differences in the transmissibility of pandemic viruses relative to zoonotic strains, and this knowledge could then be used to improve pandemic risk assessments.

## MATERIALS AND METHODS

### Cells and viruses

Recombinant A/California/07/2009 (H1N1pdm09) and A/Hong Kong/1/1968 (H3N2) viruses were generated by reverse genetics. Reverse genetics plasmids for the A/California/07/2009 (H1N1pdm09) virus were generously provided by Dr. Jesse Bloom, Fred Hutch Cancer Research Center, Seattle, WA. Reverse genetics plasmids for the A/Hong Kong/1/1968 (H3N2) virus were generated via cloning individual gene segments from the A/Hong Kong/1/1968 (H3N2) (mother clone) (NR-28620, BEI Resources). This virus has a defined passage history of three monkey cell passages and three egg passages prior to plaque purification. Each viral gene was cloned into pDP2002 following previously established methodology, and viruses were rescued in a co-culture of MDCK and 293T cells (27). After virus rescue, the virus was passaged twice in MDCK cells to generate a virus stock. MDCK cells (London Line, FR-58) were obtained through the International Reagent Resource Influenza Division, WHO Collaborating Center for Surveillance, Epidemiology and Control of Influenza, Centers for Disease Control and Prevention, Atlanta, GA, USA. MDCK cells were cultured at 37°C in 5% CO_2_ using DMEM (HyClone) media supplemented with 10% FBS (Seradigm), 4.0 mM L-glutamine (Corning), 15 mM HEPES (Corning), and 1% antibiotic and antimycotic solution (Life Technologies). 293T cells (American Type Culture Collection) were cultured under the same conditions using Opti-MEM media (Invitrogen) supplemented with 10% FBS and 1% antibiotic and antimycotic solution. When propagating the virus, the media was replaced with viral culture media consisting of Opti-MEM supplemented with 1% antibiotic and antimycotic solution, and 1 ug/mL of tosylsulfonyl phenylalanyl chloromethyl ketone-trypsin (Worthington).

### Virus titrations

The TCID_50_ of virus stocks was determined on MDCK cells seeded in 24-well plates. Serial 10-fold dilutions of each virus stock in virus culture media were overlayed on four wells of a 24-well plate and then incubated at 37°C for 96 h. At this time, plates were scored for cytopathic effect (CPE), and the TCID_50_/mL was calculated using the method of Reed and Muench (28). All virus stocks were aliquoted and stored at −80°C until use. Virus stocks were titrated a minimum of four times, and the average viral titer was used to prepare log-fold dilutions of virus for inoculation of ferrets.

For nasal wash samples, the same titration approach was used with the following modifications (16). Nasal wash samples were titrated using a combination of 96-well and 24-well plates of MDCK cells. To determine peak titers, 10-fold serial dilutions of nasal wash were added to a 96-well plate containing MDCK cells in virus culture media. Following inoculation of the MDCK cells, plates were incubated at 37°C and evaluated for CPE after 96 h. To enhance the limit of detection, 100 uL of nasal wash sample was also added to 2 wells of a 24-well plate and incubated at room temperature for an hour. The media was then replaced with virus culture media, incubated at 37°C for 4 days, and scored for CPE. The TCID_50_/mL of nasal wash was then calculated using the method of Reed and Muench (28).

### Biocontainment and animal care and use

Experiments using recombinant A/California/07/2009 (H1N1pdm09) and A/Hong Kong/1/1968 (H3N2) were performed at biosafety level 2+. All animal studies and procedures were conducted in compliance with all applicable regulations and guidelines. Male and female ferrets, aged 24–30 weeks (Triple F Farms, Sayre, PA), were used for all studies. Two male and two female DR:RC pairs (total four DR:RC pairs) were used in each transmission study. All animals were pre-screened by hemagglutination inhibition assay and confirmed seronegative for currently circulating influenza A viruses.

### Ferret transmission experiments

To evaluate airborne transmission, donor ferrets (*n* = 4/inoculation dose) were sedated by intramuscular (i.m.) injection with a combination of ketamine (30 mg/kg), xylazine (2 mg/kg), and atropine (0.05 mg/kg). Animals were intranasally inoculated with virus inoculation doses from 10^6^ to 10^0^ TCID_50_ in a 1 mL volume of Opti-MEM media (Life Technologies, CA). Following inoculation, ferrets were given an i.m. injection of the reversal agent, atipamezole (0.5 mg/kg), and were housed in individual biocontainment ferret cages (Allentown, PA). Twenty-four hours post-inoculation, each inoculated DR ferret was placed on one side of a transmission cage and paired with an RC ferret. Transmission experiments were performed using large stainless steel ventilated ferret cages (Allentown, PA) modified such that DR and RC pairs were separated by a 5 cm wide perforated offset divider (Fig. 1). Airflow on the transmission cages was calibrated to 25 air changes per hour for the entire cage.

At the time of introduction into transmission cages, and every other day for 14 days, DR ferrets were sedated by i.m. injection with ketamine (20 mg/kg), xylazine (2 mg/kg), and atropine (0.05 mg/kg), and nasal wash samples were collected by instilling a 1 mL volume of PBS into the nose and inducing sneezing onto a petri dish. An additional 1 mL of PBS was then used to rinse the dish, and nasal wash samples were aliquoted and stored at −80°C. The RC ferrets were sampled on alternating days from the DRs, and nasal wash samples were collected following the same procedure. All animals were monitored daily for clinical signs of illness, and body weight and temperatures were recorded at the time of nasal wash. At 21 days post-infection of DRs, all ferrets were deeply sedated. Blood was then collected via cardiac puncture, and animals were euthanized via overdose with sodium pentobarbital. Blood was processed to recover serum, which was stored at −80°C.

### Serology

Serum samples were evaluated for antibody titers using a hemagglutination inhibition (HI) assay. Serum was treated with receptor-destroying enzyme (Hardy Diagnostics) overnight, followed by heat inactivation at 56°C for 1 h. Sera was then diluted twofold in PBS in 96-well V-bottom plates (Corning), incubated with 8 HA units of virus/well, and then overlayed with a 0.5% suspension of turkey red blood cells (Lampire Biological Laboratories). After 35–40 min, hemagglutination was visually assessed, and the HI titer was determined as the reciprocal of the lowest serum dilution without agglutination. The limit of detection for the HI assay was a serum dilution of 1:10, and animals with HI titers at or above this limit of detection were considered seropositive.

### Statistical analyses

ID_50_ and TD_50_ were calculated with standard logistical regression on the log-transformed TCID_50_ using a generalized linear model with a logit-link function and a binomial likelihood, using the Python stats model package v0.14.4. Confidence intervals were based on standard errors from the fitted model. As nearly all 2009 H1N1 RC ferrets were infected, a reliable TD_50_ estimate and confidence intervals for 2009 H1N1 could not be obtained; calculations and regression figures for 2009 H1N1 transmission results are included in the online GitHub code repository (see Data Availability). Area under the curve analyses to assess total viral shedding, and comparisons of peak nasal wash titers were performed using GraphPad Prism (version 10.2.1). Maximal body temperature change, percent maximal weight loss, total virus shedding, and AUC were compared between DR inoculation doses for a specific virus strain using a Kruskal-Wallis test with the post hoc two-stage step-up procedure of Benjamini, Krieger, and Yekutieli test. For comparisons between virus strains at a specific dose, Mann-Whitney U tests were performed.

To determine whether the 2009 H1N1 virus is more transmissible than the 1968 H3N2, a paired nonparametric comparison was done at each inoculation dose, excluding the 10^3^ dose for which experiments on eight ferrets were done only for H3N2. This means that a total of 44 contact ferrets (not 52) were used for this comparison. To perform a basic randomization test, a test statistic of the total number of H1N1 infected contact ferrets minus the total number of H3N2 contact ferrets was defined for the true experimental results (with a value of 7 from the data). The influenza subtype labels in the data were randomly reshuffled, for each dose, among the two or three experiments done for that dose, and the test statistic was recomputed. This process was repeated 100,000 times, with 4,148 shuffled data sets having a test statistic of 7 (which was the maximum possible). A Wilcoxon signed-rank test was performed on the same dose-stratified data for 44 contact ferrets, with the two H3N2 experiments at dose 10^6^ combined as 2.5 contact ferrets infected out of four. For all statistical analyses, *P* < 0.05 was considered significant.

## Data Availability

The code used to perform linear regression of the transmission outcome data for the 2009 H1N1 and 1968 H3N2 viruses, and the vRNA sequences of the reverse genetics plasmids for these viruses, is available at https://github.com/maciekboni/influenza-ferret-transmission-td50.

## SUPPLEMENTAL MATERIAL

Table S1 jvi.01635-25-s0001.xlsx Ferret clinical scoring.

ASM does not own the copyrights to Supplemental Material that may be linked to, or accessed through, an article. The authors have granted ASM a non-exclusive, world-wide license to publish the Supplemental Material files. Please contact the corresponding author directly for reuse.
